# Challenges and Strategies for the Development and Implementation of Climate‐Informed Early Warning Systems for Vector‐Borne Diseases: A Systematic Review

**DOI:** 10.1111/tmi.70045

**Published:** 2025-09-26

**Authors:** Cong Tuan Pham, Ha Thu Nguyen, Hong H. T. C. Le, Nu Quy Linh Tran, Kien Quoc Do, Vinh Bui, Hai Phung, Dung Phung, Cordia Chu

**Affiliations:** ^1^ Centre for Environment and Population Health School of Medicine and Dentistry, Griffith University Brisbane Queensland Australia; ^2^ School of Preventive Medicine and Public Health Hanoi Medical University Hanoi Vietnam; ^3^ School of Public Health The University of Queensland Brisbane Queensland Australia; ^4^ Department of Disease Prevention and Control Pasteur Institute Ho Chi Minh City Vietnam; ^5^ Faculty of Science and Engineering Southern Cross University Gold Coast Queensland Australia

**Keywords:** challenges and strategies, climate‐informed early warning systems, vector‐borne diseases

## Abstract

**Background:**

Vector‐borne diseases, exacerbated by climate change, present an escalating global health threat, necessitating robust surveillance and climate‐informed early warning systems to predict outbreaks and enable timely interventions. This systematic review aims to synthesise the challenges and strategies involved in developing and operationalising early warning systems for vector‐borne diseases.

**Methods:**

Following PRISMA guidelines, we conducted a systematic search across multiple databases (PubMed, Web of Science, Scopus and Embase) and performed a manual search using predefined keywords up to 05 November 2024. Eleven papers were selected for the reviewing process.

**Results:**

While early warning systems show significant promise in enhancing outbreak prediction and guiding timely public health interventions, several key challenges persist. Inadequate data quality and integration—characterised by fragmented epidemiological, entomological and meteorological datasets—compromise predictive accuracy. The review also highlights gaps in stakeholder engagement and capacity building. Without comprehensive training and active collaboration among public health officials, climate scientists and data analysts, the practical application and sustainability of these systems are undermined. Enhancing data harmonisation through standardised collection processes and integration protocols is crucial for improving model reliability. The adoption of scalable, cloud‐based platforms can mitigate technical and infrastructural limitations by enabling real‐time data processing and robust computational capabilities. Strengthening interdisciplinary collaborations—bringing together experts from diverse fields—can refine predictive models and ensure that system outputs are both accurate and actionable. Furthermore, tailored capacity‐building initiatives are vital for empowering local authorities to effectively interpret and implement early warning systems' warning signals. Finally, optimising communication strategies by simplifying technical outputs and developing user‐friendly interfaces can bridge the gap between complex predictive analytics and practical decision‐making processes.

**Conclusion:**

Addressing these challenges through integrated solutions will enhance the effectiveness and sustainability of early warning systems, ultimately improving outbreak preparedness and response for vector‐borne diseases in a changing climate.

## 1 Introduction

Vector‐borne diseases (VBDs) cause over 700,000 deaths annually, with malaria and dengue being the most severe [1]. These diseases disproportionately affect tropical and low‐income regions [2, 3]. Climate change exacerbates VBDs by altering vector habitats, extending transmission seasons and expanding geographic ranges, with warming temperatures and extreme weather events creating favourable conditions for disease spread [4, 5]. By 2070, climate change could put 4.7 billion people at risk of VBDs [4, 5, 6]. Community mobilisation, vector control strategies and public health interventions are essential in reducing transmission rates of VBDs [4, 5]. Climate‐informed Early Warning Systems (EWSs), integrating climate data to predict VBD outbreaks, have shown promise in providing advanced warnings, enhancing health system readiness, triggering timely interventions and informing policy decisions [4, 7, 8, 9, 10, 11, 12, 13].

Effective EWSs, including climate‐informed EWSs for infectious diseases, consist of four key components: risk knowledge, monitoring and warning service, dissemination and communication and response capacity [14, 15, 16, 17]. Effective EWSs begin with a thorough understanding of risks, which result from the interaction of hazards and vulnerabilities in a given location [18]. These assessments, often visualised through risk maps, help prioritise early warning needs and inform prevention and response strategies [11]. A reliable monitoring and warning service is at the core of an EWS, which requires a strong scientific foundation for hazard forecasting, supported by continuous monitoring of relevant parameters [14, 15, 16, 17]. Timely and clear communication of warnings is crucial to ensuring that those at risk receive and understand the information [14]. Messages should be simple, useful and delivered through multiple communication channels to maximise reach and redundancy [19]. Pre‐established authoritative voices at regional, national and community levels enhance the credibility and effectiveness of warnings [19]. A well‐functioning EWS requires that communities not only receive warnings but also understand how to respond effectively [19]. Education, preparedness programs, and regularly tested response plans are essential for building response capacity [14, 15, 16, 17].

Thanks to a robust understanding of risks and investments in data collection, monitoring, training, and the promotion of new modelling technologies, various climate‐informed EWSs have been developed for VBDs, providing timely and accurate forecasts [12, 20]. Despite their potential, challenges remain in developing and applying these systems for VBD prevention and control [12, 20]. In particular, the communication and dissemination of warnings, as well as community response capabilities, have not received similar attention [12, 19, 20]. A comprehensive analysis is needed to guide the development of effective EWSs and ensure their sustainable integration into routine prevention and control efforts. A comprehensive synthesis of the challenges and strategies in developing and implementing these systems is notably absent from the literature. Furthermore, there is a pressing need for a comprehensive analysis that consolidates the diverse experiences and insights from various implementations of EWSs for VBD prevention and control.

This review aims to summarise the recent literature, evidence and strategies for effectively implementing climate‐informed EWSs to support sustainable VBD prevention and control. This paper identifies key gaps in EWS components, including risk knowledge, dissemination and communication, preparedness and response capabilities, which are discussed in detail hereafter. Additionally, issues related to the implementation and operationalisation of the EWSs and stakeholder engagement are also investigated.

## 2 Method

### 2.1 Literature Search

A literature search was conducted using both electronic and manual searching methods to find peer‐reviewed articles that reported on Climate‐informed EWSs for vector‐borne disease prevention and control. Published articles were searched using databases including PubMed, Web of Science, Scopus and Embase up to 05 November 2024. The search included relevant keywords and word variants for Climate, Early Warning Systems and Vector‐borne diseases. The detailed keywords and searching Boolean are presented in Table S1.

Additionally, we conducted a manual search through the reference lists of relevant papers and employed a snowballing approach by reviewing all articles that cited the included papers and examining their references to identify other pertinent materials.

### 2.2 Data Sources, Search Strategy and Eligibility Criteria

#### 2.2.1 Study Selection Criteria and Procedure

##### 2.2.1.1 Screening

Two authors screened the title, abstract and full text based on the developed review question and specific inclusion and exclusion criteria using Covidence [21]. Unsure papers were reviewed by another reviewer for the final decision.

##### 2.2.1.2 Inclusion Criteria

The study selection process aimed to identify and include articles that evaluated Climate‐Informed EWSs implemented for VBD prevention and control. First, articles must assess Climate‐Informed EWSs designed for the prevention and control of VBDs. Articles were included if they described systems that were already operational or currently being implemented. Additionally, the reported EWS must generate prospective early warning signals based on real‐world data and effectively communicate these signals to relevant stakeholders for intervention. Furthermore, the studies had to focus on human outcomes related to VBDs. Lastly, only articles published in English were reviewed.

##### 2.2.1.3 Exclusion Criteria

Articles were excluded if they failed to meet the inclusion criteria or fell into specific excluded categories. Studies describing theoretical models, computational optimisation exercises or projections for EWS that had not undergone evaluation were omitted. Research focusing on non‐human outcomes or diseases not classified as vector‐borne was also excluded. Additionally, certain types of publications, such as reviews, meta‐analyses, commentaries, editorials, case reports, letters to the editor and book chapters, were excluded due to their secondary or anecdotal nature. Articles with no accessible full text were also omitted from consideration.

#### 2.2.2 Quality Assessment

We conducted the methodological quality appraisal of the included studies. The quality of the included studies were assessed using the Mixed Methods Appraisal Tool (MMAT), version 2018 [22]. The quality of studies was also assessed based on the relevant items from the Critical Appraisal and Data Extraction for Systematic Reviews of Prediction Modelling Studies (CHARMS) checklist [23]. Studies with very low rigour, which indicated very serious flaws in the study design, were excluded.

The review was registered with PROSPERO (CRD42024562065) on 05/07/2024 and is reported according to the Preferred Reporting Items for Systematic Reviews and Meta‐Analyses (PRISMA) 2020 statement [24].

### 2.3 Data Extraction and Analysis

#### 2.3.1 Data Extraction

Data extraction was conducted using Covidence extraction form. Pilot extraction was carried out on 5%–10% of the studies to refine the extraction form and establish consensus among extractors. To ensure rigour, two independent reviewers assessed full‐text articles based on predefined criteria, conducting data extraction and analysis. Disagreements were resolved through discussion, with a third reviewer mediating if necessary.

The extracted information included comprehensive study characteristics such as study title, publication year, country of intervention, disease, objectives and study timelines. For EWSs, detailed data were collected on risk knowledge, technical monitoring, warning dissemination, response capability and methods for validating and evaluating the systems. Evidence of the effectiveness of EWSs was assessed in outbreak detection, case reduction and policy or decision‐making. Challenges in developing and implementing EWSs, including stakeholder engagement, technical limitations, risk communication, preparedness and response capacity, were documented alongside strategies to overcome these challenges.

#### 2.3.2 Data Analysis

Studies were grouped by topic, and patterns across studies were explored, considering the four elements of EWSs, and operationalisation, implementation and stakeholder engagement of the EWS. The quality of evidence was assessed to identify limitations, gaps and implications for policy and practice. Thematic analysis was used to identify patterns and codes across the included studies. A narrative synthesis, adapted from established guidance, provided a structured interpretation of findings.

## 3 Results

**FIGURE 1:**
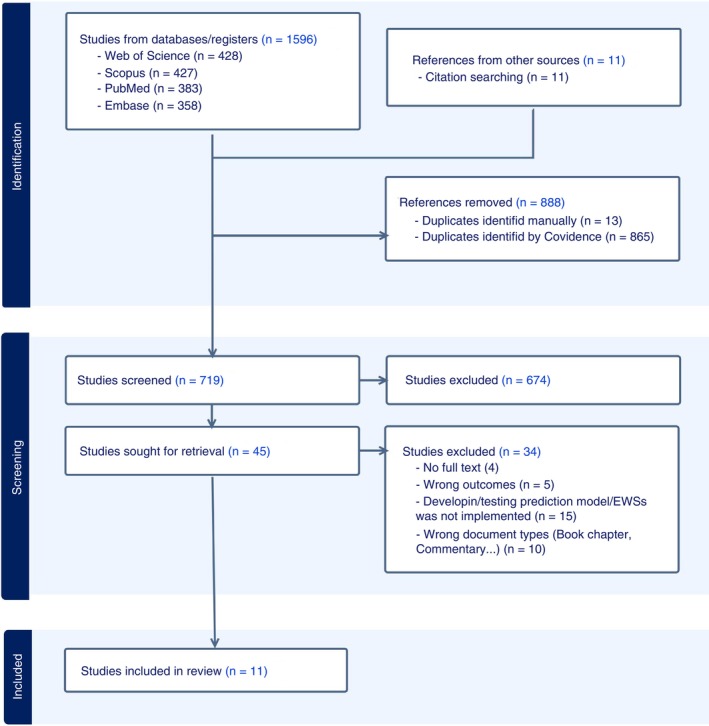
PRISMA flowchart of article selection.

Database searching recovered 719 records when duplicates were removed. After the exclusion criteria were applied, 11 papers were selected for the review process. A PRISMA flowchart documented the article selection process (Figure 1). Characteristics of included studies are summarised in Table S2.

The included studies were published between 2014 and 2023. Most of the articles (8 in 11 articles) included in the final selection reported the EWSs in low‐ and middle‐income countries in the tropical and subtropical regions including Mexico [25]; Brazil [26]; Sri Lanka [27]; Vietnam [28]; Barbados [29]; Kenya [30]; and Ethiopia [31, 32]. One article reported a dengue EWS in Singapore, a developed country in the tropical region [33]. Two articles reported EWSs that were implemented in multiple countries [34, 35].

The research studies only focus on mosquito‐borne diseases. Three articles reported malaria EWSs [30, 31, 32] and seven articles focused on dengue EWSs [25, 26, 27, 28, 29, 33, 35]. One article reported EWS for mosquito‐borne diseases, with applications in forecasting West Nile virus outbreaks and malaria epidemics [34]. Most of the studies are case study reports of the development and implementation of climate‐informed EWSs [25, 26, 27, 28, 30, 32, 33, 34]. Two studies shared experiences of co‐learning during the process of co‐creating EWSs [29, 31]. Hussain‐Alkhateeb et al. (2018) reported on the development of EWSs, based on users' recommendations [35].

This Results section presents the key findings on the challenges and strategies related to the development and implementation of EWSs for VBDs. The analysis is structured around the four core components of EWSs—risk knowledge, monitoring and warning services, dissemination and communication and response capacity. In addition, the section concludes with a focus on two critical cross‐cutting dimensions: operationalisation and stakeholder engagement. The key themes identified and analysed in the included articles are presented in Table S3.

### 3.1 Risk Knowledge

All the reported EWSs were based on an advanced understanding of VBDs' risks. Understanding local vulnerabilities and transmission patterns is crucial for designing targeted interventions and justifying investments in EWSs [25, 27]. Furthermore, studies also included the understanding of community‐based interventions in controlling disease transmission in the development and implementation of EWSs [25, 27].

Risk is defined through the integration of multiple data sources, including climatic, ecological, epidemiological and socio‐environmental factors. Historical patterns are analysed to establish baseline trends, allowing for the detection of anomalies—such as deviations from the “Endemic Channel”—that signal emerging outbreaks [35]. Climatic variabilities, particularly extreme events like El Niño, are recognised as a major trigger for outbreaks by influencing local weather conditions [30]. Spatial analysis is also crucial, with the use of risk maps, geo‐referenced case data and vector density indicators helping to pinpoint high‐risk areas [26, 28]. Furthermore, predictive modelling and EWSs are employed to forecast potential outbreaks ahead of time, while local context, including population density, community vulnerability and immunity levels, is considered vital in refining these risk assessments [32, 33].

However, many studies highlighted critical gaps in knowledge about hazards and vulnerabilities, which are essential for the development of EWSs, effective disease control and prevention strategies. Studies revealed significant gaps in risk information and highlight the uneven distribution of risk information, urging improved data collection to address community‐specific vulnerabilities [30, 34]. Stewart‐Ibarra et al. (2022) demonstrated the gaps in community‐specific risk reduction strategies, while Wimberly et al. (2022) emphasised the needs of enhancing surveillance capacity and the development of rapid data integration tools [29, 32]. Studies also highlighted gaps in risk knowledge due to significant underreporting in places like Vietnam due to passive surveillance and asymptomatic cases [28]. Additionally, Lowe et al. (2014) and Shi et al. (2016) highlighted the need for region‐specific analyses to better understand the socio‐economic, environmental and climatic factors influencing disease risks [26, 33].

The interaction between various environmental and ecological factors and disease transmission remains complex and not fully understood. Climatic variables, including El Niño‐induced weather anomalies, significantly impact conditions such as malaria epidemics in the East African Highlands, underlining the need for improved predictive models [30]. Additionally, further research should focus on understanding disease vector ecology, particularly mosquito behaviour, breeding patterns and migration dynamics, as these are crucial for improving vector control strategies and enhancing disease prevention efforts [34].

### 3.2 Monitoring and Warning Service

#### 3.2.1 Developing Monitoring and Predicting Capabilities

The Monitoring and Warning Service component received the most attention and was described in detail in the majority of studies included in this review. Effective EWSs for dengue outbreaks integrate epidemiological indicators such as the mean age of patients and circulating serotypes [30], entomological indicators including percentage positivity, Ovitrap indices, and vector egg counts [35], and meteorological indicators such as temperature, humidity and rainfall [28, 31]. Various modelling approaches, including process‐based models [30], statistical models [33, 35] and Bayesian regression [25], integrate these indicators effectively to develop prediction models. Super‐ensemble models, which aggregate multiple individual models using weighted averages, improve forecasting reliability [28]. Advancements in remote sensing [34] and spatial technologies [28] further enhance outbreak prediction. EWARS Plus and EPIDEMIA are machine learning‐based EWSs that analyse climate and disease surveillance data to provide automated, interpretable outbreak predictions [25, 32].

Evaluating prediction models is essential to ensure their reliability and applicability in outbreak forecasting. Various statistical and probabilistic techniques were employed to assess model performance [26, 28, 32]. In addition, outbreak detection and sensitivity analysis play a crucial role in ensuring that models can effectively capture epidemic signals. Sensitivity tests and Receiver Operating Characteristic (ROC) analysis optimise predictive thresholds by assessing model performance across varying outbreak scenarios [26]. Retrospective and prospective forecasting approaches further contribute to model validation. Retrospective validation involves testing models on historical data to refine predictive capabilities [26]. Prospective validation, on the other hand, assesses model performance on future data, simulating real‐time decision‐making processes [28].

Forecasting horizons play a crucial role in ensuring timely interventions for disease outbreaks. Although short‐term forecasting provides early warning signals within a few weeks, allowing rapid responses to emerging outbreaks, it may lack long‐range predictive capabilities [25, 31]. On the other hand, long‐term forecasting requires rigorous validation to ensure reliability, given the inherent uncertainties associated with extended forecasting horizons [28]. Medium‐term forecasting extends predictive insights over a one‐to‐three‐month period, facilitating strategic planning and preparedness, offering sufficient lead time for intervention strategies [26, 30, 33].

#### 3.2.2 Challenges Related to Technical Monitoring and Warning Services

##### 3.2.2.1 Data Quality and Availability

The lack of reliable, timely and high‐quality data is a limiting factor to the accuracy and effectiveness of EWSs [31]. While historical datasets are often well‐archived, real‐time data acquisition and integration remain a challenge, limiting the effectiveness of rapid response strategies and predictive modelling efforts [35]. Issues such as delayed reporting, data fragmentation, and variations in data collection methods further complicate the reliability of disease prediction models [30, 35]. Limitations in spatial and temporal data consistency (e.g., weekly temporal units or administrative changes) can hinder effective forecasting [28, 35].

Strategies to enhance data quality and accessibility include the adoption of high‐quality, globally accessible datasets with low latency [28]. Addressing inconsistencies through improved data standardisation tools and methodologies is crucial for ensuring the usability of data in predictive models [35]. Additionally, overcoming internet connectivity issues and data storage limitations is essential for ensuring seamless access to real‐time data [32]. Effective EWSs rely on the integration of multiple data sources, including epidemiological, environmental and meteorological information, to enhance predictive capabilities and ensure comprehensive risk assessments [25, 32]. Open‐access tools, such as the statistical software R, facilitate accessibility and adaptability, enabling users to analyse and visualise data efficiently [35].

##### 3.2.2.2 Modelling and Forecasting Limitations

One common issue is the accuracy and predictive capability of spatial predictions at local levels. While regional models often provide reliable forecasts, translating this information into precise district‐ or community‐level predictions remains difficult due to environmental variability and data limitations [27, 28]. In addition, disease forecasting models often rely heavily on climate variables such as temperature, humidity and rainfall. While these factors play a significant role, they do not account for all determinants of disease outbreaks, including vector indices, serotype‐specific data, population movement, interventions and socio‐economic conditions, which affect predictions [25, 27, 28].

In addition, microenvironments play a crucial role in sustaining vector populations and enabling disease transmission even when broader environmental conditions appear unsuitable. Several studies highlighted how localised ecological, infrastructural and behavioural factors can create harbourage for vectors such as mosquitoes. Githeko et al. (2018) noted that despite regional temperatures being too low to support malaria epidemics in western Kenya, localised effects of the El Niño phenomenon still triggered outbreaks, indicating the presence of conducive microhabitats [30]. Colon‐Gonzalez et al. (2021) similarly acknowledged that their models did not fully account for localised factors such as vector control interventions, infrastructure and human mobility, which can significantly alter disease risk at finer scales [28]. In small island settings, Stewart‐Ibarra et al. (2022) found that sparse data and complex micro‐ecologies limited the effectiveness of spatial analyses, underscoring the role of local environmental niches in vector persistence [29]. Additionally, Withanage et al. (2018) emphasised that daily human commuting between districts could lead to exposure in high‐risk microenvironments not reflected in surveillance data, complicating outbreak prediction [27]. These findings collectively underscore the importance of incorporating microenvironmental variability into early warning systems to enhance the accuracy and relevance of outbreak forecasts.

Continued refinement of model algorithms, combined with rigorous validation efforts, is essential for reducing prediction errors and increasing the reliability of disease warning systems. A probabilistic superensemble system that integrates multiple spatiotemporal modelling techniques can improve forecasting by providing not only point estimates but also associated uncertainty ranges. By incorporating uncertainty quantification, these probability‐based methods enhance the interpretability and reliability of predictions, thereby supporting more informed decision‐making within early warning systems [28]. Operational efficiency can also be improved by optimising data processing speed and user interface design to streamline model application in real‐world scenarios [35]. Additionally, leveraging environmental datasets, such as vegetation indices and satellite‐based rainfall estimates, can improve the early detection of disease risks [28]. Iterative improvements, guided by stakeholder feedback and real‐world testing, have allowed systems to evolve and remain relevant in changing climatic and epidemiological conditions [28, 34].

##### 3.2.2.3 Challenges in Monitoring, Equipment, Technology and Infrastructure

Technological and infrastructure‐related constraints significantly affect the efficiency of disease EWSs. Equipment failures, unstable internet connectivity and gaps in meteorological station networks disrupt data collection and analysis processes [30, 32]. Furthermore, traditional client‐based systems, such as EASTWeb, EPIDEMIA and Google Earth Engine (GEE), often face challenges in processing and storing large datasets [32, 34]. In contrast, cloud‐based solutions offer potential improvements in computational efficiency and data accessibility [34]. Transitioning to more scalable cloud environments may enhance performance and flexibility in handling extensive datasets [34]. Innovative solutions, such as low‐bandwidth system designs, can help overcome these challenges [25, 32].

### 3.3 Dissemination and Communication

The success of EWSs depends on a well‐integrated approach that ensures warnings reach their intended audiences, are easily understood, and contain actionable and locally relevant information. All 11 EWSs reported in this review were designed to inform government agencies and public health authorities, enabling timely actions [25, 26, 27, 28, 29, 30, 31, 32, 33, 34, 35]. While some systems also communicated warnings to at‐risk communities, often through simplified messages—such as press reports regarding the potential dengue risk situation in Brazil [26], or public messaging on mosquito breeding prevention in Barbados [29]—the primary intended users and decision‐makers remained institutional stakeholders. To ensure early warnings reach the target audience, it is crucial to utilise effective communication channels that integrate existing institutions and media platforms [30]. Integrating operational partners in communication ensures timely updates for health departments [33]. However, many challenges remain in the timely dissemination of understandable warnings to the public health officials and authorities.

#### 3.3.1 Community Awareness, Perception and Trust

A critical challenge in disseminating warnings is the lack of community awareness about risks and EWSs. Communities and local health officials do not fully understand the potential threats they face, leading to low engagement and limited cooperation in response efforts [29]. Additionally, cultural beliefs and social norms shape how communities perceive risk information, sometimes creating resistance to adopting recommended protective measures [25]. Trust in EWSs is also critical for their effectiveness, yet it can be easily eroded by perceived inaccuracies or false alarms [29]. When preventive actions successfully mitigate risks, the absence of a visible outbreak event may lead to scepticism about the validity of warnings, discouraging future compliance [29].

Effective communication strategies are essential for building public trust and promoting the adoption of EWSs. Engaging media outlets to share information about system functionality and benefits increases public awareness and encourages community participation [30]. Government agencies, like the Ministry of Health and the Kenya Meteorological Department, play key roles in building trust in the warnings [30]. Public health messages via epidemic forecast reports, climate risk maps, climate service platforms and bulletins effectively reach health decision‐makers during dengue outbreaks [29]. Establishing feedback loops between stakeholders facilitates continuous communication, allowing for real‐time updates and improvements based on user experiences [28].

#### 3.3.2 Challenges in Risk Communication

Warnings often contain complex technical information that can be difficult for non‐expert audiences to understand. The use of scientific terminology, probabilistic forecasts, and statistical uncertainties can create confusion and reduce the effectiveness of warnings [33, 35]. Translating probabilistic data into actionable messages requires decision support tools, such as risk matrices, that communicate certainty levels and urgency more intuitively [26]. In the case of predictive models for outbreaks like dengue, the inclusion of multiple variables and covariates can further complicate interpretation, making it necessary to simplify messaging while maintaining scientific accuracy [33]. In addition, customisation of warnings to local needs enhances their effectiveness. Users could download data and reports tailored to specific epidemiological and environmental variables, ensuring the information was relevant to their context [31]. The value of combining probabilistic forecasts with urgency levels helps stakeholders prioritise their responses based on the severity of potential outbreaks [29].

Moreover, generic alerts without specific instructions on how to respond can lead to uncertainty and inaction among affected communities [35]. Developing risk communication frameworks that include localised impact assessments, such as identifying “hot spots” for targeted interventions, can enhance the relevance and applicability of warnings [33]. Warnings with actionable insights ensure that recipients can take appropriate measures to mitigate risks. Warnings included recommendations such as restocking medical supplies and deploying insecticide‐treated nets in high‐risk areas [30]. Risk maps further enhanced response efforts by identifying high‐probability outbreak zones, enabling targeted interventions [28]. Similarly, EWARS Plus quantified outbreak rates and certainty intervals, providing valuable information for public health decision‐makers [25].

Uncertainty in predictive models poses a significant challenge in risk communication. Forecasting systems often struggle to convey the limitations of long‐term predictions, leading to either overconfidence or scepticism among decision‐makers [26, 33]. Clearly articulating the uncertainties and confidence levels in forecasts is crucial to ensuring informed decision‐making and efficient resource allocation. Decision‐makers need to be equipped with tools that help interpret forecast reliability, reducing the risk of misinterpretation and suboptimal responses [26].

#### 3.3.3 Technical and Technological Barriers

Ensuring that warnings reach the health authorities and target populations remains a persistent challenge, particularly in remote or underserved areas. Limited infrastructure, technological barriers and socio‐economic disparities can impede access to crucial information [30, 34]. The reliance on advanced technological tools for warning dissemination can create additional barriers, particularly for non‐technical users or those with limited access to digital platforms [32]. Systems that require real‐time data streaming may be vulnerable to disruptions during crises such as pandemics, undermining their reliability [29]. Additionally, technical failures, such as equipment breakdowns and data availability issues, can interrupt warning dissemination, necessitating the development of resilient systems capable of maintaining functionality under adverse conditions [30]. While automating EWSs can improve sustainability, it also introduces complexities that may require ongoing technical support and capacity‐building efforts [29].

User‐friendly interfaces play a significant role in facilitating the dissemination and communication of warning signals. Transitioning from STATA to the R interface reduced complexity and improved user engagement [35]. Similarly, simplified interfaces in Google Earth Engine and REACH enhanced data access and visualisation for users with varying technical expertise [32]. In addition to interface design, clear visualisations contribute to better comprehension of risk information. Studies reported using colour saturation techniques to visualise probabilistic forecasts, effectively conveying uncertainty and risk levels [26], and incorporating charts and maps in EPIDEMIA reports, providing clear summaries of outbreak risks and trends for end‐users [31].

### 3.4 Response Capability

Effective EWSs rely on their ability to support proactive responses, enhance community preparedness and ensure effective implementation through continuous updates and practice. Among the reviewed studies, only 6 out of 11 reported on the response capability and preparedness of EWSs [26, 29, 30, 32, 33, 35].

The identification of high‐risk areas is crucial for targeted interventions and pre‐positioning of critical resources. Hotspot mapping tools facilitate the visualisation of disease risk, enabling policymakers and health agencies to deploy resources efficiently and supporting early preventive measures [29]. Githeko et al. (2018) demonstrated how proactive measures such as restocking drugs, diagnostic supplies and insecticide‐treated nets can significantly improve outbreak response [30]. Similarly, Shi et al. (2016) highlighted the value of forecasting disease peaks to support strategic resource planning and early risk communication [33].

The implementation of structured response plans is fundamental to effective disease control. Hussain‐Alkhateeb et al. (2018) advocated for national guidelines and standardised protocols that outline staged responses to dengue outbreaks, ensuring that communities and healthcare providers have clear instructions on how to act during epidemics [35]. Additionally, Lowe et al. (2014) highlighted the benefits of shifting control strategies based on early warnings, underscoring the importance of collaboration among public health specialists, climate scientists and disease modellers [26].

However, significant challenges related to preparedness and response capabilities remain. In many regions, early warning frameworks lack formalised partnerships and clear mandates, making it difficult to integrate climate‐health considerations into public health decision‐making [29]. Additionally, prioritisation of high‐risk areas (“hot spots”) and efficient resource allocation remain key challenges in optimising disease surveillance and response strategies [26, 35]. Strengthening collaboration between governmental agencies, research institutions, and local communities is crucial to ensuring that preparedness plans remain relevant and actionable in dynamic disease landscapes.

Another significant issue is the complexity of standardising response protocols across diverse local contexts while ensuring adequate resource allocation [25]. Addressing these barriers requires robust policy frameworks, sustained financial investments and institutional commitment to integrating EWS into public health operations.

### 3.5 Operationalising Early Warning Systems and Stakeholder Engagement

#### 3.5.1 Operationalising EWSs for Disease Prediction and Public Health Response

Studies demonstrated a wide range of successes and practical contributions to disease control and interventions through the implementation of EWSs. Successful adaptation of EWSs to diverse environments highlights their flexibility and relevance [29, 32]. EWSs have been incorporated into national and regional health strategies, enhancing disease surveillance and response [25, 33]. Implementation often began with pilot testing in multiple regions before full‐scale deployment, as seen with EPIDEMIA in Ethiopia and EASTWeb in studies on West Nile Virus [34, 35]. Dengue EWSs have supported public health programs in Singapore by improving resource planning and facilitating early risk communication [33]. Climate‐informed health planning is becoming more prevalent, as seen in Barbados' regional Health Climatic Bulletin [29]. Simplified tools and automated systems help lower technical barriers, allowing non‐specialists to adopt EWSs more readily in health operations [34].

Public health messages informed by EWSs outputs played a pivotal role in resource allocation, risk communication and disease prevention efforts [29, 33]. Forecasts were used to guide interventions such as restocking essential medicines, deploying diagnostic tools, hospital bed management and distributing insecticide‐treated nets [30]. Early warnings supported public awareness campaigns, facilitated hospital resource planning and enhanced targeted control measures such as vector management [33]. Early warnings enable proactive interventions, leading to significant reductions in disease incidence as demonstrated in Singapore's dengue prevention program [33] and Kenya's malaria control measures [30].

#### 3.5.2 Stakeholder Engagement for Developing and Implementing EWSs

The successful development and implementation of EWSs depend on collaborative design, continuous communication, customisation and local adaptation. The involvement of diverse groups of stakeholders in EWSs development and implementation enhances the system's relevance and adaptability, addressing both scientific and operational needs [28, 29]. Furthermore, local ownership plays a crucial role in ensuring the long‐term sustainability of these systems. By tailoring EWSs to local needs and resource availability, developers can create solutions that are practical and resilient in the face of changing environmental and health challenges [25].

High levels of stakeholder approval and acceptance are critical for the successful implementation of EWSs [29, 30, 31, 35]. Studies emphasised that securing buy‐in from key stakeholders, including government agencies, health departments and local communities, facilitates system uptake and integration into existing public health frameworks [30]. Effective communication strategies and transparent decision‐making processes have been identified as essential in gaining trust and ensuring long‐term sustainability [30].

Co‐creation and collaborative design are essential strategies for ensuring that EWSs meet user needs while incorporating scientific rigour [29, 31, 35]. Regular stakeholder workshops and consultations provide an avenue for co‐developing system requirements and integrating user feedback into the design process [29, 31]. Workshops dedicated to co‐design enable public health professionals, researchers and software engineers to collaboratively develop user interfaces, data upload processes and system security measures. An iterative development approach, wherein stakeholders contribute feedback at different stages, fosters a dynamic system that evolves based on practical needs and real‐world applications [35]. These interactions help identify system requirements, including user access protocols, data integration strategies and automated reporting mechanisms. By incorporating stakeholder input from the outset, EWS developers can ensure that the final product is user‐friendly, functional, and aligned with institutional workflows.

Institutionalising EWSs through formal agreements and governance structures strengthens long‐term sustainability and operational efficiency. Establishing Memorandums of Understanding and joint work plans between key organisations formalises partnerships and ensures clear roles and responsibilities [29]. Steering committees composed of representatives from government agencies, non‐government organisations and research institutions provide oversight and strategic direction, ensuring that EWS implementation aligns with national preparedness priorities [28].

#### 3.5.3 Experience and Lessons Learned From Operationalising EWSs

Despite advancements, several challenges persist in operationalising EWSs. One of the key challenges in operationalising lies in the integration of the EWSs into national health strategies and the development of local capacity for long‐term management [30]. Embedding these systems into public health frameworks ensures institutional continuity and access to necessary resources [25, 29]. Ensuring that EWSs align with existing workflows and local conditions is essential for successful integration into public health decision‐making processes [25, 32]. Ongoing evaluation and updates based on new data and user feedback further enhance the system's relevance and functionality [28].

Another major challenge in implementing EWSs is the shortage of professionals skilled in software engineering, statistical modelling and risk communication [29]. Specialised training programs can bridge these gaps and enhance system efficiency. Capacity‐building initiatives, such as stakeholder training and knowledge‐sharing workshops, improve preparedness by fostering collaboration among researchers, policymakers and health officials [29]. Training in data management, statistical analysis and risk communication empowers stakeholders to make informed decisions [35]. Furthermore, the lack of standardised training for public health officials leads to inconsistent responses, weakening disease prevention. Structured training programs enhance decision‐making and promote ownership among local authorities, ensuring long‐term sustainability [25, 35].

Sustained funding is a major challenge for scaling EWSs long‐term. Many rely on short‐term grants, causing disruptions when funding ends [28, 29]. Limited resources hinder the transition from pilots to sustainable systems, affecting data collection, model development and response measures [28, 29]. To ensure EWS functionality and impact, long‐term funding mechanisms and stronger policy commitments are essential.

## 4 Discussion

### 4.1 Key Challenges and Strategies for Development and Implementation of EWSs

This systematic review is the first to comprehensively examine the challenges and strategies involved in developing and applying climate‐informed EWSs for vector‐borne diseases. The review examines 11 journal articles according to the four components of EWSs, stakeholder engagement and the operationalisation of EWSs. The integration of EWS outputs into public health interventions has demonstrated significant benefits, including improved resource allocation, risk communication and proactive disease prevention. The review findings also highlight several interrelated challenges to the development and implementation of EWSs.

First, many studies consistently reported that fragmented data—resulting from inconsistent collection methods and variable quality across epidemiological, entomological and meteorological sources—substantially limit predictive accuracy [28, 30, 31, 34, 35].

In response to these challenges, the literature proposes several promising strategies. The adoption of scalable, cloud‐based platforms is one such strategy, enabling the processing of large data volumes in real time and thereby enhancing forecast accuracy and timeliness. The need for a sound scientific basis for predicting and forecasting hazards, as well as the importance of appropriate equipment for data handling and prediction modelling, is a critical factor in establishing effective warning services [14]. Moreover, implementing standardised data collection protocols along with advanced statistical techniques, such as probabilistic forecasting and super‐ensemble modelling, can help mitigate inherent uncertainties in climate and disease data. The World Meteorological Organization calls for integrating climate and health data to enable more nuanced risk assessments [36].

Moreover, technical limitations—such as outdated software, limited real‐time processing and inadequate automation—undermine system reliability. Infrastructural constraints hinder the seamless integration of diverse datasets necessary for robust predictive modelling [37, 38]. This finding is shared by other systematic reviews [39]. These challenges are particularly pronounced in low‐resource settings, where functional climate information services, technological infrastructure, and reliable internet connectivity are often lacking [40].

Third, risk communication remains problematic. The importance of disseminating warnings in an efficient and timely manner, in a format suited to user needs, has been emphasised in recent literature [37, 41]. Warnings often rely on technical language that is inaccessible to non‐expert stakeholders, eroding community trust and delaying timely responses. Significant challenges persist, particularly the erosion of trust in EWSs due to perceived inaccuracies or false alarms [29]. Moreover, the inherent uncertainty in long‐term forecasts can lead to either overconfidence or scepticism among decision‐makers [29]. This issue is further compounded by the prevalence of infodemics and health misinformation, which can undermine the credibility of EWS alerts [42]. This aligns with the need for clear, accessible language and culturally appropriate messaging to ensure community trust and timely responses. Establishing continuous feedback loops between stakeholders is essential to refining communication strategies and ensuring that warnings include actionable insights [28].

Fourth, the operationalisation of EWSs is frequently challenged by a shortage of professionals skilled in software engineering, statistical modelling and risk communication, as well as by the lack of standardised training for public health officials [29, 35]. A second key barrier was insufficient training for practitioners who would use the tools [25, 35]. Capacity‐building initiatives—through targeted training programs and stakeholder workshops—ensure that public health officials and community leaders are well‐equipped to interpret and act on EWS outputs [25, 35]. For example, the World Health Organization supports countries in implementing the Early Warning and Response System (EWARS), which includes capacity‐building components to ensure effective use of the system [43].

Fifth, insufficient stakeholder engagement—from data scientists and public health officials to community members—continues to limit the potential impact of EWSs. Recent research highlighted the need to involve non‐state actors, including businesses, in EWS development and implementation [44, 45]. A multi‐sectoral approach can significantly enhance the reach and effectiveness of EWSs, particularly at the local level [38, 45]. Similarly, a landscape mapping study, which included interviews with researchers and policy stakeholders, identified insufficient stakeholder engagement as a key barrier to the adoption of climate‐informed CSID models as forecasting tools by public health practitioners [46]. The limited collaboration among modellers, tool developers (e.g., software engineers), and decision‐makers impedes the translation of models into practical tools for public health use.

Collaborative design and co‐creation processes involving diverse groups—ranging from local authorities to community representatives—ensure that the systems are tailored to meet both scientific standards and practical needs [29, 31]. Institutionalising EWSs through formal agreements and governance structures enhances their long‐term sustainability and operational efficiency. Systems that effectively integrated inputs from health agencies, academic institutions, meteorological services, and international organisations demonstrated improved functionality and precision. Strengthening interdisciplinary collaboration, fostering co‐creation with local stakeholders, and synergies between both producers and users of EWSs are equally vital [16]. Engaging communities in both the design and dissemination of early warnings improves the clarity and relevance of risk communication, builds trust and facilitates prompt public health action.

Finally, a significant challenge in implementing effective EWSs is the complexity of standardising response protocols across diverse local contexts while ensuring adequate resource allocation. Standardised response protocols that can be effectively implemented across different regions are often unavailable, which limits response capacity [26, 35]. Strengthening policy and regulatory frameworks is also essential to prioritise early warning and response mechanisms, alongside restructuring institutional architecture to foster greater inclusivity and stakeholder participation in decision‐making processes [44]. While many studies emphasised the importance of EWSs, fewer address the critical role of regular simulation exercises in validating response plans [17]. Conducting consistent drills and evaluations is essential for identifying weaknesses and refining strategies to enhance system effectiveness. Without sufficient practice and iterative improvement, even the most well‐designed response plans risk being ineffective in real‐world scenarios.

### 4.2 Limitations and Gaps of the Previous Studies

Our systematic review reveals significant limitations in the current literature regarding the development and operationalisation of EWSs for VBDs. Despite the proliferation of climate‐informed forecasting models, only a small subset of studies has progressed from model development to practical implementation. This review highlighted that, while numerous studies have explored forecasting models, only 11 articles reported on the operationalisation of VBD EWSs. A related review further underscores this gap by noting that among 30 tools focused on VBDs, merely 20 were presented as accessible products, and only about one‐quarter of these featured interfaces tailored for decision makers [46].

A major shortcoming across the reviewed studies is the absence of rigorous evaluations of EWS implementation and operational effectiveness. Most publications were case reports focusing on the development and implementation phases without robust outcome assessments [25, 26, 27, 28, 32, 34]. In several instances, although reductions in VBD outbreaks or case numbers were reported, the studies were unable to definitively attribute these improvements to the use of EWSs [30, 33]. Githeko et al. (2018) noted that even with widespread use of insecticide‐treated nets and effective antimalarials, climate variability continued to trigger epidemics [30]. This lack of causal linkage calls into question the actual impact of these systems and underscores the need for more comprehensive impact evaluations. Future research should be directed towards longitudinal studies that assess the long‐term operational success of EWSs, as well as comparative evaluations across diverse epidemiological settings.

Additionally, economic evaluation is another area where the current literature falls short. There is a dearth of compelling evidence on the cost‐effectiveness of EWSs, which is critical for persuading public health authorities to invest in these tools. Without clear data demonstrating that EWSs can effectively reduce the incidence of VBDs at a reasonable cost, the case for their broader adoption remains unconvincing [35]. This gap not only hampers the refinement of existing systems but also limits strategic planning and investment in scaling up these tools for broader application [35]. Exploring the economic implications of scaling up these systems is essential to ensure sustainable funding and long‐term viability.

Beyond the scope of our formal inclusion criteria, we noted a striking paucity of studies evaluating the economic benefits of vector‐borne disease EWSs. This gap likely derives from the methodological challenges inherent in such analyses: when an early warning system successfully averts an outbreak, there is no “event” against which to compare costs and quantifying the value of avoided cases, hospitalisations, and productivity losses requires sophisticated modelling of counterfactual scenarios [47, 48]. Moreover, data on health‐system expenditures and indirect economic impacts are often fragmented or unavailable, further complicating cost‐benefit assessments [47, 49]. Future research should therefore develop and apply standardised frameworks and data collection protocols to capture the economic value of EWS interventions [48, 49].

In addition to evaluation challenges, the integration of EWSs with public health response capacities remains problematic. The review identified that only 6 of the 11 studies addressed the development of response capacity [26, 29, 30, 32, 33, 35]. Given that the success of an EWS is inherently tied to the ability of public health systems to act on the generated alerts, insufficient emphasis on response capacity represents a critical barrier to the effective use of these systems in VBD prevention and control. It is clear that developing robust response frameworks and ensuring that alerts translate into timely and appropriate interventions is essential for realising the potential benefits of EWSs.

Furthermore, the reviewed literature predominantly focuses on mosquito‐borne diseases, particularly dengue [25, 26, 27, 28, 29, 33, 35] and malaria [30, 31, 32]. This narrow scope limits the generalisability of the findings and highlights the need for further research on EWSs for a broader range of VBDs.

## 5 Conclusion

This systematic review highlights both the potential and the challenges of developing and implementing climate‐informed EWSs for VBDs. While EWSs have demonstrated significant benefits—such as improved risk communication, proactive disease prevention and optimised resource allocation—persistent barriers hinder their full integration into public health practice. Key challenges include fragmented data sources, technical limitations, inadequate risk communication strategies, insufficient workforce capacity and limited stakeholder engagement. Addressing these issues requires robust interdisciplinary collaboration, standardised protocols and sustained investment in human resources and infrastructure. Long‐term success depends on stakeholder engagement and capacity building. Collaborative design with communities, public health authorities and experts, along with structured training programs and integration into national health strategies, will secure sustained funding and operational sustainability for EWSs.

Additionally, gaps in the current literature—particularly the lack of rigorous evaluations of EWS impact, cost‐effectiveness and integration with response mechanisms—underscore the need for further research. Future efforts should prioritise operational assessments, economic analyses and comparative studies across different epidemiological contexts. Strengthening policy frameworks and fostering community participation will be essential in ensuring that EWSs become reliable, actionable tools for VBD prevention and control. By addressing these challenges, EWSs can play a critical role in enhancing public health resilience in the face of climate‐driven disease risks.

## Conflicts of Interest

The authors declare no conflicts of interest.

## Supporting information


**Data S1:** tmi70045‐sup‐0001‐Tables.docx.
