# Supplementary material for: Challenges and Strategies for the Development and Implementation of Climate‐Informed Early Warning Systems for Vector‐Borne Diseases: A Systematic Review
Source: Trop Med Int Health. 2025 Sep 26;31(1):10–21. doi: 10.1111/tmi.70045 (PMC12775892; doi:10.1111/tmi.70045)
Supplement: Supplementary file 1 — Data S1: tmi70045‐sup‐0001‐Tables.docx. [file TMI-31-10-s001.docx]

# **Supplemental Table #1. Search term and Search strategy**

| **Category** | **Search term** |
| --- | --- |
| Climate  AND | Climate OR Weather OR rainfall OR humidity OR ambient temperature |
| Early Warning Systems  AND | early warning system OR EWS OR early warning and response system OR EWARS OR prediction tool OR early warning |
| Vector-born disease | vector-borne disease OR VBD OR VBDs OR tick-borne disease OR mosquito-borne disease OR mosquito-transmitted disease OR arboviral disease OR flea-borne disease OR louse-borne disease OR mite-borne disease OR sandfly-borne disease OR dengue OR malaria OR zika OR chikungunya OR yellow fever |

| **Database** | **Search Boolean** |
| --- | --- |
| PubMed | (((((climate) OR (weather)) OR (((rainfall) OR (rain)) OR (humidity))) OR (ambient temperature))  AND  ("early warning system*" [All fields] OR "EWS" [All fields] OR "early warning and response system" [All fields] OR "EWARS" [All fields] OR prediction tool* [All fields] OR "early warning" [All fields])  AND  ((((((((((((((((vector-borne disease) OR (tick-borne disease)) OR (mosquito-borne disease)) OR (flea-borne disease)) OR (louse-borne disease)) OR (mite-borne disease)) OR (sandfly-borne disease)) OR (dengue)) OR (VBD)) OR (VBDs)) OR (malaria)) OR (zika)) OR (chikungunya)) OR (yellow fever)) OR (mosquito-transmitted disease)) OR ("arboviral disease*"))) |
| Scopus | ( ( ( ( ( climate ) OR ( weather ) ) OR ( ( ( rainfall ) OR ( rain ) ) OR ( humidity ) ) ) OR ( "ambient temperature" ) )  AND  ( "early warning system*" OR ews OR "early warning and response system" OR ewars OR "prediction tool*" OR "early warning" )  AND  ( ( ( ( ( ( ( ( ( ( ( ( ( ( ( ( "vector-borne disease" ) OR ( "tick-borne disease" ) ) OR ( "mosquito-borne disease" ) ) OR ( "flea-borne disease" ) ) OR ( "louse-borne disease" ) ) OR ( "mite-borne disease" ) ) OR ( "sandfly-borne disease" ) ) OR ( dengue ) ) OR ( vbd ) ) OR ( vbds ) ) OR ( malaria ) ) OR ( zika ) ) OR ( chikungunya ) ) OR ( "yellow fever" ) ) OR ( "mosquito-transmitted disease" ) ) OR ( "arboviral disease*" ) ) ) |
| Embase | 'climate'/exp OR climate OR 'weather'/exp OR weather OR 'rainfall'/exp OR rainfall OR 'rain'/exp OR rain OR 'humidity'/exp OR humidity OR 'environmental temperature'/exp OR 'environmental temperature'  AND  'early warning system*' OR ews OR 'early warning and response system' OR ewars OR 'prediction tool*' OR 'early warning'  AND  'vector-borne disease' OR 'tick-borne disease' OR 'mosquito-borne disease' OR 'flea-borne disease' OR 'louse-borne disease' OR 'mite-borne disease' OR 'sandfly-borne disease' OR dengue OR vbd OR vbds OR malaria OR zika OR chikungunya OR 'yellow fever' OR 'mosquito borne disease' OR 'arbovirus infection' |
| Web of Science | (((((climate ) OR (weather )) OR (((rainfall ) OR (rain )) OR (humidity ))) OR ("ambient temperature" ))  AND  ("early warning system*" OR EWS OR "early warning and response system" OR ewart OR "prediction tool*" OR "early warning")  AND  (((((((((((((((("vector-borne disease" ) OR ("tick-borne disease" )) OR ("mosquito-borne disease" )) OR ("flea-borne disease" )) OR ("louse-borne disease" )) OR ("mite-borne disease" )) OR ("sandfly-borne disease" )) OR (dengue )) OR (VBD )) OR (vads )) OR (malaria )) OR (zika )) OR (chikungunya )) OR ("yellow fever" )) OR ("mosquito-transmitted disease" )) OR ("arboviral disease*" ))) |

# **Supplemental Table #2. Included articles**

| **Study reference** | **Country of intervention** | **Region** | **Disease** | **Study aim** | **Implementation/operationalise** |
| --- | --- | --- | --- | --- | --- |
| 1. Githeko, A. et al. The launch and operation of the malaria epidemic early warning system in Kenya. Science Publishing Group Journal 1 (2018). | Kenya | Kisii and Kakamega County, two highland sites | Malaria | Presents results of the malaria epidemic early warning system’s predictions and data interpretations from sites in western Kenya highlands. | The final product was launched with over 95% approval. It was implemented at the Kenya Meteorological Department in 2011. A meteorologist ran the models monthly, and the data were assessed by the Ministry of Health's malaria control division. Extra supplies were provided when an epidemic risk was identified. |
| 2. Liu, Y. et al. Software to facilitate remote sensing data access for disease early warning systems. ENVIRONMENTAL MODELLING & SOFTWARE 74, 247-257 (2015). | United States and Ethiopia | West Nile virus outbreaks in the United States and malaria epidemics in the Ethiopian highlands | Mosquito-borne diseases | Implement and test early warning systems for case studies of two mosquito-borne diseases in different social and ecological contexts. | EASTWeb software was used to create environmental databases for early warning systems for West Nile virus and malaria. The software collected environmental data to generate predictions for WNV and malaria, with varying accuracy at regional and county levels. |
| 3. Merkord, C. L. et al. Integrating malaria surveillance with climate data for outbreak detection and forecasting: the EPIDEMIA system. Malaria Journal 16, 89 (2017). | Ethiopia | Amhara region | Malaria | Documents the design and implementation of EPIDEMIA, a prototype system for integrating malaria surveillance with environmental monitoring data to generate operational forecasts of malaria outbreak risk. | The EPIDEMIA system collected malaria surveillance data from 2012 to 2016, showing significant improvements in data accuracy and completeness. Environmental data were also collected and processed for analysis. |
| 4. Hussain-Alkhateeb, L. et al. Early warning and response system (EWARS) for dengue outbreaks: Recent advancements towards widespread applications in critical settings. PLoS ONE 13 (2018). | Brazil, Malaysia, and Mexico | 30 health districts in Brazil, Malaysia, and Mexico | Dengue | Summarizes recent adaptation efforts guided by local health managers' experience and further statistical analyses that underpin recent modifications. | The first generation of the EWARS tool identified indicators predicting outbreaks. It was tested in Brazil, Malaysia, and Mexico, with feedback collected through questionnaires. |
| 5. Colón-González, F. J. et al. Probabilistic seasonal dengue forecasting in Vietnam: A modelling study using superensembles. PLoS Med 18, e1003542 (2021). | Vietnam | N/A | Dengue | Produces results at the province level and assesses the predictive ability of the D-MOSS system consistently across Vietnam. | A superensemble model was used to generate dengue forecasts for May to October 2020. The predictions were slightly more accurate than the baseline model, with little variability between ensemble members. |
| 6. Lowe, R. et al. Dengue outlook for the World Cup in Brazil: an early warning model framework driven by real-time seasonal climate forecasts. Lancet Infect Dis 14, 619-626 (2014). | Brazil | 553 microregions of Brazil, with risk level warnings for the 12 cities | Dengue | Provides probabilistic forecasts of dengue risk for the 553 microregions of Brazil with risk-level warnings for the 12 cities where the World Cup matches will be played. | Dengue risk warnings were issued for World Cup host cities in 2014, with varying risk levels. The forecasting system performed well for previous years. |
| 7. Shi, Y. et al. Three-Month Real-Time Dengue Forecast Models: An Early Warning System for Outbreak Alerts and Policy Decision Support in Singapore. Environ Health Perspect 124, 1369-1375 (2016). | Singapore | Singapore | Dengue | Forecasts the evolution of dengue epidemics in Singapore to provide early warning of outbreaks and facilitate the public health response. | The forecasting tool became part of Singapore's dengue control program, providing weekly updates and guiding public health interventions during the 2013 epidemic. |
| 8. Wimberly, M. C., Nekorchuk, D. M. & Kankanala, R. R. Cloud-based applications for accessing satellite Earth observations to support malaria early warning. Sci Data 9, 208 (2022). | Ethiopia | 852 districts in Ethiopia | Malaria | Facilitates regular access to satellite data in support of the EPIDEMIA malaria early warning project in Ethiopia. | The REACH application, part of the EPIDEMIA system, used climate data to model malaria and generate forecasts. Machine learning algorithms were used to predict weekly malaria cases in Ethiopia. |
| 9. Stewart-Ibarra, A. M. et al. Co-learning during the co-creation of a dengue early warning system for the health sector in Barbados. BMJ Glob Health 7 (2022). | Barbados | Barbados | Dengue | Shares experiences of co-learning during the process of co-creating a dengue EWS for the health sector in Barbados, discussing barriers to implementation and key opportunities. | A pilot dengue prediction tool was created for Barbados, incorporating climate information to predict outbreaks three months in advance. The tool helped inform public health planning and interventions. |
| 10. Sanchez Tejeda, G. et al. Early warning and response system for dengue outbreaks: Moving from research to operational implementation in Mexico. PLOS Glob Public Health 3, e0001691 (2023). | Mexico | N/A | Dengue | Describes the implementation process of EWARS in Mexico, demonstrating benefits, threats, and opportunities of integrating EWARS into existing national surveillance programs. | EWARS was validated for dengue outbreaks in Mexico, showing successful outbreak prevention with timely responses. Mexico incorporated EWARS into its national dengue surveillance platform in 2018. |
| 11. Withanage, G. P., Viswakula, S. D., Nilmini Silva Gunawardena, Y. I. & Hapugoda, M. D. A forecasting model for dengue incidence in the District of Gampaha, Sri Lanka. Parasit Vectors 11, 262 (2018). | Sri Lanka | District of Gampaha | Dengue | Analyzes spatial and seasonal distribution of dengue incidence and proposes a simple and precise dengue early warning system based on local meteorological factors using time series regression methods. | Models were developed to forecast dengue cases in Gampaha, Sri Lanka, from 2015 to 2017. The models showed normal distribution of residuals and captured five out of nine outbreaks during the testing period. |

# **Supplemental Table #3. Key themes that mention in the included articles**

| **Key themes** | **Articles that mentioned the themes** | | | | | | | | | | |
| --- | --- | --- | --- | --- | --- | --- | --- | --- | --- | --- | --- |
|  | **(1)** | **(2)** | **(3)** | **(4)** | **(5)** | **(6)** | **(7)** | **(8)** | **(9)** | **(10)** | **(11)** |
| **1. Risk Knowledge** |  |  |  |  |  |  |  |  |  |  |  |
| Advancing the understanding of vector-borne disease (VBD) risks is essential for the development of effective early warning systems (EWSs). | x | x | x | x |  | x |  | x | x |  | x |
| Significant gaps remain in knowledge about hazards and vulnerabilities, both of which are critical for designing robust EWSs. |  | x |  | x | x | x |  | x | x | x |  |
| The interaction between various environmental and ecological factors and disease transmission is complex and not yet fully understood. |  |  |  |  |  | x |  |  |  | x |  |
| **2. Monitoring and Warning Service** |  |  |  |  |  |  |  |  |  |  |  |
| Developing and evaluating prediction models is essential to ensure their reliability and applicability for outbreak forecasting. | x | x | x | x | x | x | x | x | x | x | x |
| Forecasting horizons play a crucial role in enabling timely public health interventions. | x | x |  | x |  | x | x |  | x |  |  |
| Data quality and availability remain significant challenges for technical monitoring and warning services. |  |  |  | x |  | x | x |  |  | x | x |
| Modelling and forecasting limitations include: Limited accuracy and predictive capability at local spatial levels; and Inability of models to incorporate all determinants of disease outbreaks, such as vector indices, serotype-specific data, population movement, interventions, and socio-economic conditions. | x |  | x | x | x | x |  |  |  | x | x |
| Challenges in monitoring also stem from inadequate equipment, outdated technology, and insufficient infrastructure, all of which hinder the efficiency of EWSs. | x |  |  |  |  | x |  | x |  | x |  |
| **3. Dissemination and Communication** |  |  |  |  |  |  |  |  |  |  |  |
| Effective dissemination and communication are essential to inform government agencies, public health authorities, and communities. | x | x | x | x | x | x | x | x | x | x | x |
| A key challenge is the lack of community awareness and trust in the risks and the EWSs themselves. | x |  |  | x | x | x |  |  |  |  |  |
| Risk communication challenges include: Use of complex scientific terminology, probabilistic forecasts, and statistical uncertainties; Difficulty in translating probabilistic data into actionable messages; and the need to customize warnings to local contexts to enhance relevance and effectiveness. |  | x |  |  | x | x | x |  | x |  | x |
| Technical and technological barriers for dissemination and communication of EWSs' warning include: Limited infrastructure and access to digital platforms; Technological failures; and Socio-economic disparities affecting communication reach and comprehension. |  | x |  |  | x | x | x |  |  | x | x |
| **4. Response Capability** |  |  |  |  |  |  |  |  |  |  |  |
| Building the response capability of EWSs is vital for enabling proactive action, enhancing community preparedness, and ensuring effective implementation. |  | x |  |  | x | x |  | x | x |  | x |
| Structured response plans, such as national guidelines and standardized protocols, are essential. |  | x |  |  |  |  |  |  |  |  | x |
| Key challenges for building response capacity based on EWSs include: Lack of formalized partnerships and clearly defined mandates; and Limited preparedness and capacity at both institutional and community levels. |  | x |  |  | x |  |  |  |  |  | x |
| **5. Operationalizing Early Warning Systems and Stakeholder Engagement** |  |  |  |  |  |  |  |  |  |  |  |
| **5.1 Operationalizing EWSs for Disease Prediction and Public Health Response** |  |  |  |  |  |  |  |  |  |  |  |
| EWSs have demonstrated success and practical contributions to disease control and intervention strategies. | x |  |  |  | x |  |  | x | x | x | x |
| Public health messages based on EWS outputs have played a pivotal role in resource allocation, risk communication, and disease prevention. |  |  |  |  | x | x |  |  | x |  |  |
| **5.2 Stakeholder Engagement for Developing and Implementing EWSs** |  |  |  |  |  |  |  |  |  |  |  |
| The successful development and implementation of EWSs require collaborative design, ongoing communication, and adaptation to local contexts. | x |  |  | x | x |  |  |  |  |  |  |
| High levels of stakeholder acceptance and support are crucial. |  |  |  |  | x | x | x |  |  |  | x |
| Co-creation and participatory approaches help ensure that EWSs meet end-user needs. |  |  |  | x | x |  |  |  |  |  | x |
| Institutionalizing EWSs through formal agreements and governance frameworks strengthens long-term sustainability and operational efficiency. |  |  |  | x | x |  |  |  |  |  |  |
| **5.3 Experiences and Lessons Learned from Operationalizing EWSs** |  |  |  |  |  |  |  |  |  |  |  |
| Integration of EWSs into national health strategies and the development of local capacity are key to long-term success. | x |  |  | x | x | x |  | x |  |  |  |
| A shortage of professionals skilled in software engineering, statistical modelling, and risk communication, along with a lack of standardized training for public health staff, presents a major challenge. | x |  |  |  | x |  |  |  |  |  | x |
| Sustained funding remains a critical barrier to the long-term scaling and effectiveness of EWSs. |  |  |  | x | x |  |  |  |  |  |  |

**Reference:**

1. Sanchez Tejeda G, Benitez Valladares D, Correa Morales F, Toledo Cisneros J, Espinoza Tamarindo BE, Hussain-Alkhateeb L, et al. Early warning and response system for dengue outbreaks: Moving from research to operational implementation in Mexico. PLOS Glob Public Health. 2023;3(9):e0001691.

2. Lowe R, Barcellos C, Coelho CA, Bailey TC, Coelho GE, Graham R, et al. Dengue outlook for the World Cup in Brazil: an early warning model framework driven by real-time seasonal climate forecasts. Lancet Infect Dis. 2014;14(7):619-26.

3. Withanage GP, Viswakula SD, Nilmini Silva Gunawardena YI, Hapugoda MD. A forecasting model for dengue incidence in the District of Gampaha, Sri Lanka. Parasit Vectors. 2018;11(1):262.

4. Colón-González FJ, Soares Bastos L, Hofmann B, Hopkin A, Harpham Q, Crocker T, et al. Probabilistic seasonal dengue forecasting in Vietnam: A modelling study using superensembles. PLoS Med. 2021;18(3):e1003542.

5. Stewart-Ibarra AM, Rollock L, Best S, Brown T, Diaz AR, Dunbar W, et al. Co-learning during the co-creation of a dengue early warning system for the health sector in Barbados. BMJ Glob Health. 2022;7(1).

6. Githeko A, Ototo E, Muange P, Zhou GZ, Yan G, Sang J. The launch and operation of the malaria epidemic early warning system in Kenya. Science Publishing Group Journal. 2018;1(2).

7. Merkord CL, Liu Y, Mihretie A, Gebrehiwot T, Awoke W, Bayabil E, et al. Integrating malaria surveillance with climate data for outbreak detection and forecasting: the EPIDEMIA system. Malaria Journal. 2017;16(1):89.

8. Wimberly MC, Nekorchuk DM, Kankanala RR. Cloud-based applications for accessing satellite Earth observations to support malaria early warning. Sci Data. 2022;9(1):208.

9. Shi Y, Liu X, Kok SY, Rajarethinam J, Liang S, Yap G, et al. Three-Month Real-Time Dengue Forecast Models: An Early Warning System for Outbreak Alerts and Policy Decision Support in Singapore. Environ Health Perspect. 2016;124(9):1369-75.

10. Liu Y, Hu JM, Snell-Feikema I, VanBemmel MS, Lamsal A, Wimberly MC. Software to facilitate remote sensing data access for disease early warning systems. ENVIRONMENTAL MODELLING & SOFTWARE. 2015;74:247-57.

11. Hussain-Alkhateeb L, Kroeger A, Olliaro P, Rocklöv J, Sewe MO, Tejeda G, et al. Early warning and response system (EWARS) for dengue outbreaks: Recent advancements towards widespread applications in critical settings. PLoS ONE. 2018;13(5).
